# Socioeconomic factors contributing to under-five mortality in sub-Saharan Africa: a decomposition analysis

**DOI:** 10.1186/s12889-019-7111-8

**Published:** 2019-06-14

**Authors:** Carine Van Malderen, Agbessi Amouzou, Aluisio J. D. Barros, Bruno Masquelier, Herman Van Oyen, Niko Speybroeck

**Affiliations:** 1 Institute of Health and Society (IRSS), Université catholique de Louvain, Brussels, Belgium; 2 Johns Hopkins Bloomberg School of Public Health, 615 N. Wolfe St, Baltimore, MD 21205 USA; 3 Universidade Federal de Pelotas, Pelotas, Brazil; 4 Centre de Recherche en Démographie, Université catholique de Louvain, Louvain-la-Neuve, Belgium; 5 Institut National d’Etudes Démographiques (INED), Paris, France; 6 Department of Public Health and Surveillance, Sciensano, Brussels, Belgium; 7 Department of Public Health, Ghent University, Ghent, Belgium

**Keywords:** Under-five mortality, Sub-Saharan Africa, Poisson regression, Gini decomposition, Socioeconomic inequalities

## Abstract

**Background:**

In sub-Saharan Africa, socioeconomic factors such as place of residence, mother’s educational level, or household wealth, are strongly associated with risk factors of under-five mortality (U5M) such as health behavior or exposure to diseases and injuries. The aim of the study was to assess the relative contribution of four known socioeconomic factors to the variability in U5M in sub-Saharan countries.

**Methods:**

The study was based on birth histories from the Demographic and Health Surveys conducted in 32 sub-Saharan countries in 2010–2016. The relative contribution of sex of the child, place of residence, mother’s educational level, and household wealth to the variability in U5M was assessed using a regression-based decomposition of a Gini-type index.

**Results:**

The Gini index – measuring the variability in U5M related to the four socioeconomic factors – varied from 0.006 (95%CI: 0.001–0.010) in Liberia 2013 to 0.034 (95%CI: 0.029–0.039) in Côte d’Ivoire 2011/12. The main contributors to the Gini index (with a relative contribution higher than 25%) were different across countries: mother’s educational level in 13 countries, sex of the child in 12 countries, household wealth in 11 countries, and place of residence in 8 countries (in some countries, more than one main contributor was identified).

**Conclusions:**

Factors related to socioeconomic status exert varied effects on the variability in U5M in sub-Saharan African countries. The findings provide evidence in support of prioritizing intersectoral interventions aiming at improving child survival in all subgroups of a population.

## Background

The under-5 mortality rate (U5MR), the probability of dying before 5 years of age (per 1000 live births), is a key global indicator of child health [1], and one of the most important measures of global health [2]. Previously targeted in the fourth Millennium Development Goal (MDG), U5MR now appears in the third Sustainable Development Goal (SDG3) [3], aiming to reduce under-5 mortality to at least as low as 25 deaths per 1000 live births in all countries by 2030. Though the global U5MR dropped from 93 deaths per 1000 live births in 1990 to 39 in 2017, the highest rates are still seen in sub-Saharan Africa, with an U5MR of 76 deaths per 1000 live births in 2017, leading to 2.7 million deaths in the region. A vast majority of these deaths are amenable to health care and prevention, as the leading causes of death among children under age five include preterm birth complications, pneumonia, intrapartum-related complications, diarrhea and malaria [4, 5].

Even if U5MR has declined in most sub-Saharan African countries, substantial inequalities between sub-groups of the population still exist within countries [6]. Population sub-groups may be defined by different dimensions such as place of residence, sex of children, ethnicity, and maternal factors such as educational level, occupation or income [7]. These socioeconomic factors are included in the Mosley and Chen conceptual framework as the distal determinants of child mortality [7]. In this framework, socioeconomic determinants operate at three levels (the community, the household, and the individual) and affect mortality through proximate determinants such as maternal factors, environmental contamination, nutrient deficiency, injury, and personal illness control.

The magnitude of socioeconomic inequalities in U5MR may be assessed by studying each socioeconomic factor separately, using the concentration index, and income or wealth are among the most widely studied [8–10]. However, the concentration index measuring wealth-related inequalities in U5MR was not significant in several sub-Saharan countries [8, 11], an observation calling for further research on other dimensions of socioeconomic inequalities in U5MR.

Identifying the larger socioeconomic gaps in U5MR across a population may be done by assessing the relative contribution of several socioeconomic factors to the variability in U5MR, using a multivariate regression model and additional decomposition techniques [12].

Taking a set of four socioeconomic factors representative of the multiple dimensions of a society’s stratification in U5MR, the aim of the study was to identify which one(s) contributed the most to the variability in U5MR in sub-Saharan African countries.

## Methods

### Conceptual framework

Four factors were selected as proxies for the main socioeconomic determinants introduced by Mosley & Chen [13]: place of residence for the community level (ecological setting, political economy and health system), household wealth for the household level (goods and services such as food, housing, transportation, financial access to care), mother’s educational attainment for the individual level (mother’s choices and skills in health care practices), and sex of the child also for the individual level (differential feeding and medical care practices). However, the gender of the child may represent both gender discrimination and a biological disadvantage [14]. These four factors are the main socioeconomic factors used to describe U5MR by population subgroups at the international level [6, 10, 14, 15].

### Data

Data from 32 Sub-Saharan African countries with a standard Demographic and Health Survey (DHS) completed in 2010–2016 (the most recent available at the time of the analysis, in July 2018) were used. Details on survey sampling, data collection and data processing are available on the DHS Program website [16]. In total, the study gathered data from 366,960 children obtained from the interview of 248,732 mothers.

The outcome variable was under-five death. This information was obtained from the birth history of interviewed females aged 15 to 49 [16]. The analysis was restricted to the last 5 years preceding the survey to limit the time gap between the event and the collection of socioeconomic information. The socioeconomic factors investigated were: sex of the child, place of residence (urban or rural), mother’s educational level (lower than primary, primary and above), and household wealth (poorest/middle tertiles versus the highest tertile). Household wealth tertiles were derived from the wealth index provided with the DHS data, constructed from several household assets (type of flooring, water supply, sanitation facilities, electricity, persons per sleeping room, ownership of agricultural land, domestic servant, and other assets). U5MR was calculated in each wealth tertile, then the tertiles “poorest” and “middle”, showing similar levels of U5MR in a majority of countries, were pooled to have the same number of categories for the four variables, hence avoiding any bias overestimating the contribution of variables with more categories. The proportion of missing values for the included variables was lower than 0.03% in all countries.

### Data analysis

Distinct individual level analyses were carried out for the 32 included countries. U5MR was estimated in each population sub-group with the synthetic cohort probability method employed in DHS [17]. Differences in U5MR between subgroups were tested with a bootstrap technique.

The relative contribution of each factor to the variability in U5MR was made using a regression-based decomposition of a Gini index, described below.

A multivariate Poisson regression model (with exposure time as offset) was used. Multi-collinearity was checked and no variance inflation factor was greater than 10. Analyses were weighted (using weights available in the DHS datasets), accounting for clustering (with cluster as the primary sampling unit and household as the secondary sampling unit) and for stratification (with region as strata). The significance threshold was set up at 5%.

Variability in the obtained individuals’ predicted death rates was assessed with a Gini index (G), and decomposed using Wagstaff’s method [18–21]. G is defined as twice the covariance of the health variable (here predicted death rates) and the person’s fractional rank in the distribution of health, divided by the mean level of health. As the mean of the predicted death rates was negative, G ranged between − 1 (maximum variability) and 0 (no variability), but for the description of the variability in all countries, the sign was reversed so higher values mean more variability. A factor’s relative contribution to the Gini index is the product of its elasticity ($\frac{b\overline{x}}{\overline{y}}$, where *b* is the factor’s regression coefficient, $\overline{x}$ its mean and $\overline{y}$ the mean of the predicted death rates) and its concentration index (a Gini-type measure of its unequal distribution in the population ranked by predicted death rate), divided by the overall G. Factors’ concentration indexes ranged from - 1 (the factor is more concentrated among the lower values of the health variable) to 1 (the factor is more concentrated among the higher values of the health variable), 0 meaning equal distribution in the population. The four factors’ relative contributions were presented in percent, adding up to 100%. A factor was defined as a main contributor if it contributed to at least 25% to the variability in U5M.

RGui (R version 3.4.0., The R foundation for Statistical Computing) and Stata 14.0 (calculation of U5M) were used for the data analysis (an overview of the R code is provided in Appendix 1).

## Results

### Under-five mortality rate (U5MR) versus variability in U5MR in the 32 countries

**Fig. 1:**
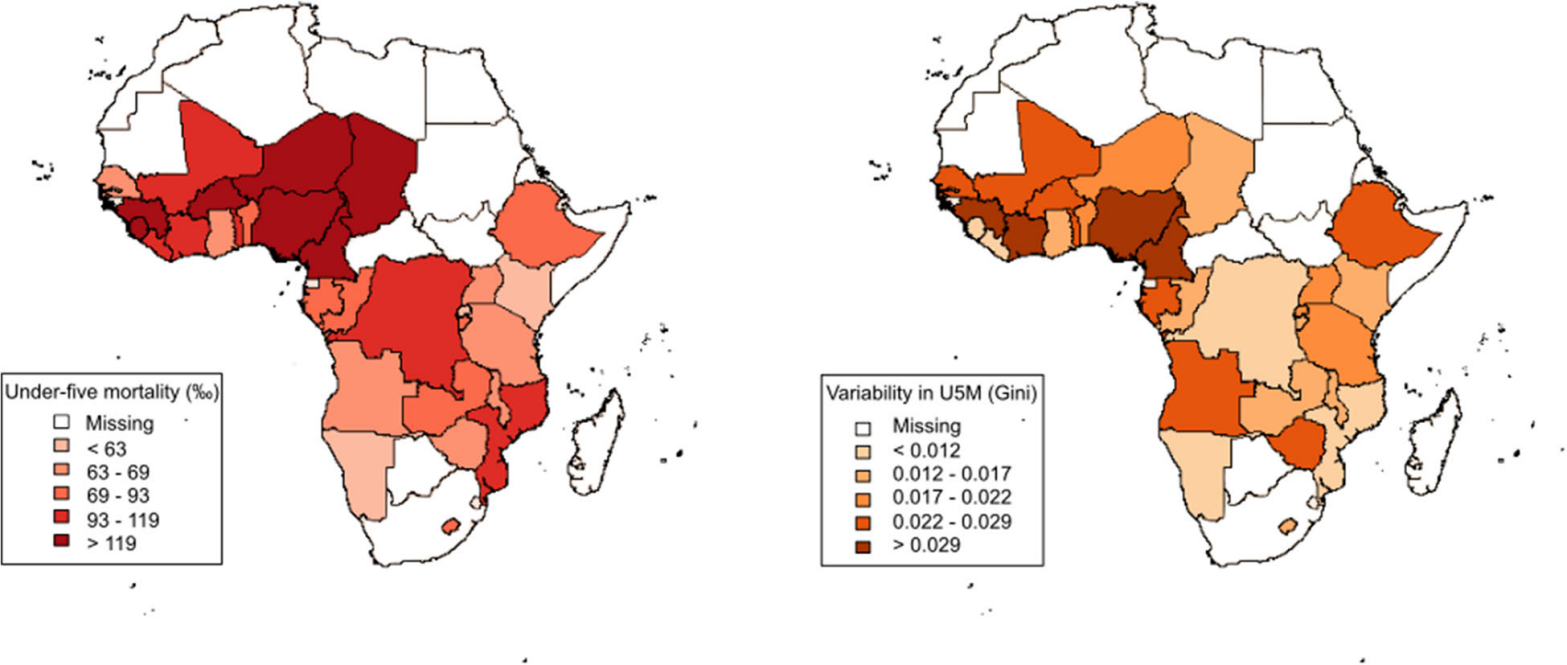
Under-five mortality rates (U5MR, left) versus variability in U5MR measured by a Gini index (right) in the 32 countries, Demographic and Health Surveys 2010–2016 (0–5 years prior to each survey)

U5MR ranged from 49 [95%CI:42;57] deaths per 1000 live births in Rwanda 2014/15 to 152 [95%CI:138;166] in Sierra Leone 2013 (Fig. 1). The Gini index, assessing the variability in U5MR related to the four factors – sex of the child, place of residence, mother’s education and household wealth, varied from 0.006 (95%CI: 0.001–0.010) in Liberia 2013 to 0.034 (95%CI: 0.029–0.039) in Côte d’Ivoire 2011/12. No correlation could be identified between U5MR and the Gini indexes (Pearson’s correlation coefficient r = 0.10, *p* = 0.59). Table 1 shows U5MR in all countries, at national level and by population subgroup. U5MR was significantly lower in females compared to males in 9 countries, in urban compared to rural areas in 12 countries (the contrary was observed in Tanzania 2015/16 where U5MR was higher in urban areas), in children born to mothers with at least a primary education compared to children born to mothers without any formal education in 19 countries, and in the richest households compared to the poorest of middle wealth households in 17 countries.

**Table 1:** Under-five mortality (‰) by determinant in the 32 countries, Demographic and Health Surveys 2010–2015

			Sex of the child			Place of residence			Mother’s education			Household wealth		
	n	Total	Male	Female	*p*	Rural	Urban	*p*	None	Primary+	*p*	Poorest/Middle	Richest	*p*
Western Africa														
Burkina Faso 2010	15,044	128	139	117	0,016	137	80	< 0.001	133	61	< 0.001	144	96	< 0.001
Senegal 2010–11	12,326	66	73	60	0,12	82	41	< 0.001	71	33	< 0.001	76	47	< 0.001
Benin 2011–12	13,407	72	77	66	0,11	82	56	0,001	76	47	< 0.001	81	52	< 0.001
Cote d’Ivoire 2011–12	7776	112	145	79	< 0.001	113	111	0,84	119	80	0,004	111	115	0,80
Guinea 2012	7039	123	135	109	0,032	140	74	< 0.001	130	68	< 0.001	148	71	< 0.001
Niger 2012	12,558	133	148	118	0,032	143	65	< 0.001	135	84	0,001	143	112	0,026
Gambia 2013	8088	52	54	50	0,70	50	54	0,67	60	33	0,003	52	50	0,82
Mali 2012–13	10,326	102	122	80	< 0.001	110	64	< 0.001	107	45	< 0.001	119	67	< 0.001
Liberia 2013	7606	96	104	88	0,32	91	101	0,51	101	83	0,34	102	83	0,34
Nigeria 2013	31,482	121	129	113	0,001	143	80	< 0.001	151	82	< 0.001	146	71	< 0.001
Sierra Leone 2013	11,938	152	163	141	0,063	153	150	0,89	157	131	0,032	153	151	0,86
Togo 2013–14	6979	93	90	97	0,49	101	79	0,10	106	61	0,001	104	71	0,004
Ghana 2014	5884	63	76	50	0,12	73	51	0,18	87	44	0,030	72	46	0,045
Central Africa														
Cameroon 2011	11,732	128	128	128	0,99	149	98	< 0.001	167	82	< 0.001	147	89	< 0.001
Congo 2011–12	9329	70	78	63	0,22	64	74	0,28	75	68	0,50	71	69	0,93
Gabon 2012	6067	89	112	63	0,098	96	87	0,57	92	88	0,89	89	88	0,95
DRCongo 2013–14	18,716	107	113	102	0,25	113	95	0,096	118	93	0,007	114	93	0,058
Chad 2014–15	18,623	138	149	126	0,001	137	140	0,84	139	129	0,53	145	125	0,043
Eastern Africa														
Mozambique 2011	11,102	94	101	87	0,092	97	88	0,36	98	76	0,060	97	88	0,38
Comoros 2012	3149	51	52	50	0,89	58	33	0,024	60	35	0,060	51	51	>0,99
Kenya 2014	20,964	54	58	49	0,13	50	60	0,095	58	51	0,18	56	49	0,25
Rwanda 2014–15	7856	49	57	42	0,030	49	49	0,99	57	34	< 0.001	55	38	0,015
Malawi 2015–16	17,286	63	71	55	0,083	65	51	0,20	66	55	0,15	69	52	0,038
Tanzania 2015–16	10,233	68	72	64	0,20	62	85	0,009	71	66	0,63	67	69	0,75
Ethiopia 2016	10,641	71	81	61	0,087	74	46	0,024	73	48	0,13	73	69	0,65
Uganda 2016	15,522	65	72	58	0,015	69	49	< 0.001	75	50	< 0.001	71	53	0,002
Burundi 2016–17	13,192	77	82	72	0,13	79	57	0,070	81	64	0,028	84	62	0,018
Southern Africa														
Namibia 2013	5046	59	60	58	0,87	63	55	0,47	63	58	0,65	62	54	0,44
Zambia 2013–14	13,457	70	77	63	0,075	71	68	0,60	77	63	0,040	73	65	0,19
Lesotho 2014	3138	86	78	97	0,32	91	77	0,38	108	80	0,11	88	84	0,84
Zimbabwe 2015	6132	66	75	58	0,059	74	49	0,006	87	63	0,095	77	46	< 0.001
Angola 2015–16	14,322	68	73	63	0,18	85	57	< 0.001	79	49	< 0.001	80	44	< 0.001

U5M and *p*-values were calculated using the synthetic cohort probability method (“syncmrates” function in Stata)

### Factors associated with U5MR in the multivariate analyses, and their relative contribution to the variability in U5MR

**Fig. 2:**
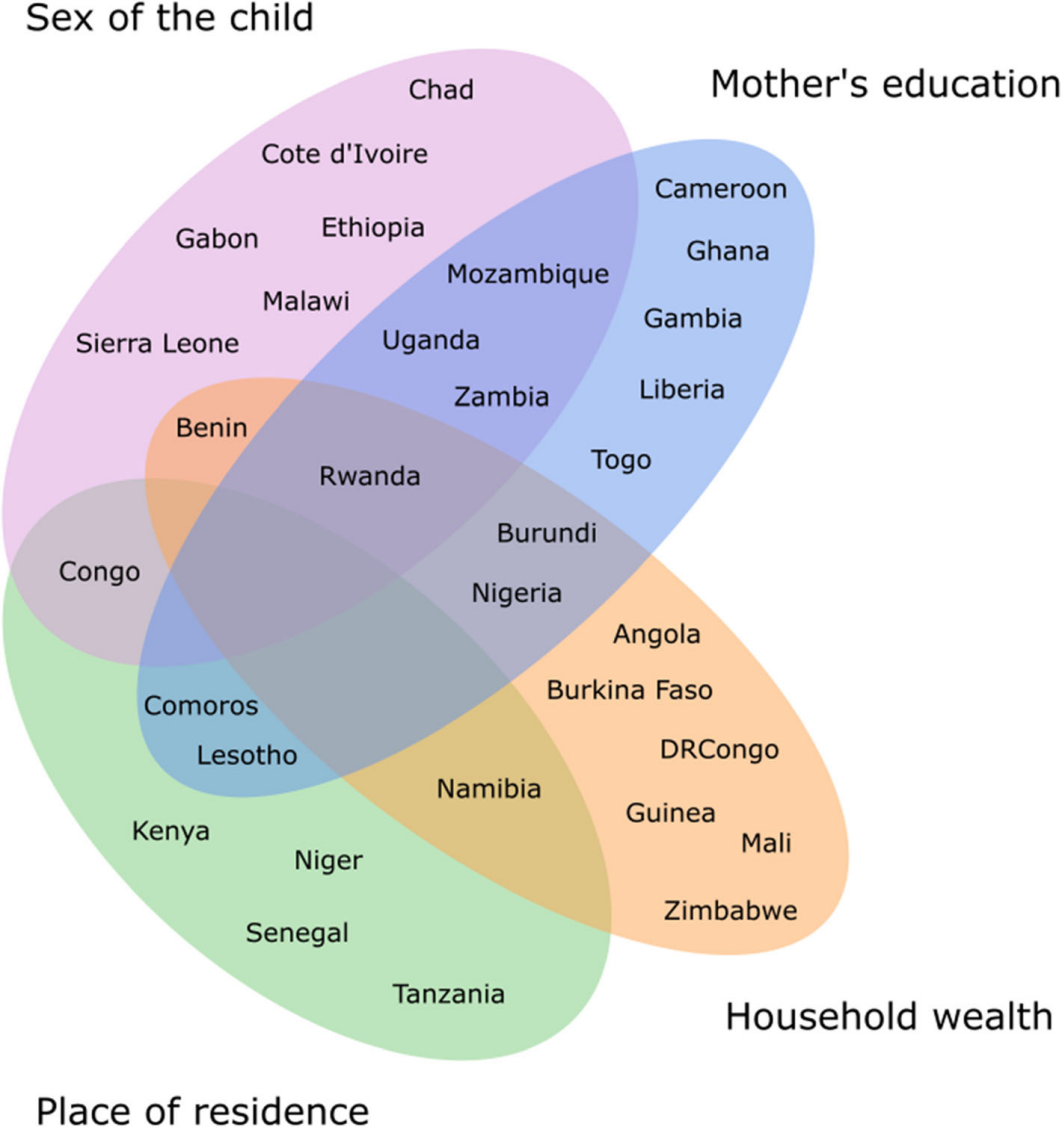
Classification of the 32 countries according to the main contributors to the variability in U5MR. A factor was defined as a main contributor if its contribution to the variability in U5MR exceeded 25% according to the regression-based decomposition of the Gini index (Table 2). The Venn diagram was developed using the R function “venn.diagram”

Table 2 shows all components of the Gini index decomposition: the overall Gini index, the mean of the predicted death rates (obtained with the multivariate Poisson regression model), variables’ means, regression coefficients, and concentration indexes. The relative contribution of a factor is calculated as the product of its mean, its regression coefficient, and its concentration index, divided by the mean of the predicted death rates, then by the overall Gini index. For instance, the relative contribution of sex of the child in the 2011–2012 DHS in Benin equals 0.48*(− 0.20)*(− 0.29) / − 6.34 / -0.017 = 26%. In Fig. 2, countries were classified according to the factor(s) which contributed to more than 25% of the variability in U5M. As the relative contributions add up to 100% and we consider 4 different factors, a contribution exceeding 25% for one factor suggests the existence of a socioeconomic gap in U5MR more marked than for the other factors.

**Table 2:** Means, regression coefficient and relative contribution (%) of the selected determinants in the 32 sub-Saharan countries, Demographic and Health Surveys 2010–2015

				Sex of the child				Place of residence				Mother’s education				Household wealth			
				Female				Urban				Primary+				Richest			
	n	G	mean(y)	mean	b	c	%_Gini_	mean	b	c	%_Gini_	mean	b	c	%_Gini_	mean	b	c	%_Gini_
Western Africa																			
Senegal 2010–11	12,326	-,026	−6,37	,49	**-,23**	-,24	16	,38	**-,44**	-,61	60	,12	-,30	-,73	15	,33	-,09	-,50	9
Burkina Faso 2010	15,039	-,024	−5,77	,49	**-,13**	-,23	10	,17	-,22	-,78	20	,07	-,30	-,84	13	,33	**-,36**	-,66	56
Benin 2011–12	13,407	-,017	-6,34	,48	**-,20**	-,29	26	,40	,00	-,24	0	,15	-,06	-,59	5	,33	**-,34**	-,67	69
Cote d’Ivoire 2011–12	7776	-,034	−5,83	,50	**-,63**	-,50	75	,37	-,29	-,27	14	,18	-,30	-,44	11	,33	,24	-,16	0
Guinea 2012	7039	-,029	−5,78	,49	-,16	-,24	11	,26	-,04	-,67	4	,12	-,34	-,75	18	,33	**-,52**	-,67	68
Niger 2012	12,537	-,017	-5,85	,49	-,10	-,39	18	,13	**-,73**	-,87	82	,06	,00	-,23	0	,33	,05	-,05	0
Mali 2012–13	10,326	-,028	−6,04	,48	**-,31**	-,29	25	,19	-,13	-,71	10	,10	-,28	-,70	11	,33	**-,41**	-,66	53
Gambia 2013	8088	-,018	−6,55	,49	-,03	-,12	1	,48	,25	,13	13	,32	**-,46**	-,68	85	,33	,04	,11	1
Liberia 2013	7606	-,006	−6,00	,49	-,02	-,22	8	,50	,10	,12	19	,32	-,09	-,53	48	,33	-,09	-,29	25
Nigeria 2013	31,482	-,032	−5,77	,50	**-,15**	-,15	6	,35	**-,23**	-,57	25	,45	**-,27**	-,51	33	,33	**-,30**	-,65	36
Sierra Leone 2013	11,938	-,007	−5,46	,50	**-,13**	-,50	92	,26	,02	,38	7	,21	,01	,26	2	,33	-,01	,08	0
Togo 2013–14	6979	-,025	−6,16	,50	**-,22**	-,21	15	,36	-,17	-,51	21	,30	**-,39**	-,66	52	,33	-,10	-,55	13
Ghana 2014	5884	-,016	−6,44	,48	-,16	-,27	20	,45	-,01	-,12	1	,58	-,34	-,42	80	,33	,04	-,11	0
Central Africa																			
Cameroon 2011	11,732	-,030	−5,79	,51	-,04	-,11	1	,42	-,11	-,45	12	,48	**-,44**	-,52	63	,33	-,20	-,59	23
Congo 2011–12	9329	-,015	−6,31	,50	-,23	-,32	38	,61	,29	,17	32	,71	-,08	-,10	6	,33	-,25	-,28	24
Gabon 2012	6067	-,022	−6,29	,48	**-,42**	-,49	71	,84	-,17	-,07	7	,74	-,25	-,15	20	,33	,05	,11	1
Chad 2014–15	18,623	-,013	−5,69	,49	**-,24**	-,51	78	,20	,19	,24	12	,15	-,04	-,30	2	,33	**-,16**	-,10	7
DRCongo 2013–14	18,716	-,011	−5,93	,50	-,08	-,27	15	,31	,04	-,45	0	,48	-,05	-,31	11	,33	**-,24**	-,67	74
Eastern Africa																			
Mozambique 2011	11,102	-,010	−5,91	,49	-,13	-,37	42	,28	,00	-,41	1	,17	-,15	-,81	35	,33	-,09	-,43	22
Comoros 2012	3138	-,029	−6,57	,49	-,10	-,13	3	,27	**-,58**	-,70	56	,35	-,42	-,53	41	,33	,31	-,03	0
Kenya 2014	20,964	-,012	−6,52	,49	-,12	-,33	25	,36	**,33**	,45	69	,60	-,09	-,06	5	,33	-,22	-,02	2
Rwanda 2014–15	7856	-,017	−6,63	,50	**-,23**	-,41	42	,17	,00	-,40	0	,34	-,23	-,47	32	,33	-,22	-,41	26
Malawi 2015–16	17,286	-,014	−6,42	,50	**-,28**	-,50	75	,13	,05	-,14	0	,29	,05	,02	0	,33	-,21	-,33	25
Tanzania 2015–16	10,233	-,019	−6,31	,49	-,19	-,31	22	,27	**,48**	,73	73	,66	,08	,13	5	,33	-,13	,30	0
Ethiopia 2016	10,638	-,022	−6,31	,48	**-,44**	-,52	78	,11	-,36	-,46	13	,09	-,06	-,27	1	,33	,24	,14	8
Uganda 2016	15,521	-,019	−6,37	,50	**-,28**	-,34	38	,21	-,16	-,48	13	,41	**-,29**	-,50	49	,33	,04	-,18	0
Burundi 2016–17	13,189	-,015	−6,23	,49	-,13	-,22	15	,09	,12	-,46	0	,25	-,18	-,63	29	,33	**-,26**	-,63	56
Southern Africa																			
Namibia 2013	5046	-,010	−6,45	,51	-,06	-,17	8	,49	-,12	-,48	43	,77	-,02	-,12	2	,33	-,14	-,65	47
Zambia 2013–14	13,446	-,012	−6,27	,49	**-,21**	-,50	70	,34	,07	,05	1	,49	-,15	-,26	27	,33	-,02	-,08	1
Lesotho 2014	3138	-,012	−5,97	,51	-,01	-,11	1	,29	-,17	-,60	38	,79	-,29	-,21	62	,33	,09	-,21	0
Zimbabwe 2015	6132	-,026	−6,29	,51	-,25	-,29	20	,32	,16	-,57	0	,86	-,24	-,09	10	,33	**-,61**	-,66	70
Angola 2015–16	14,319	-,027	−6,36	,50	**-,29**	-,28	23	,60	-,23	-,30	23	,37	-,06	-,41	5	,33	**-,38**	-,67	48

G: Gini index; mean(y): mean of the predicted (ln) death rates; mean: mean of the dummy variable (coded 0/1), corresponds to the proportion of the factor in the country; b: regression coefficient from a multivariate Poisson model (in bold if significant); %_Gini_: relative factor’s contribution calculated with the regression-based decomposition of the Gini index_h_; A few negative contributions were restricted to 0

#### Sex of the child

After adjustment for the three other covariates, the U5MR was significantly lower in girls than in boys in 16 countries (Table 2). Among the 12 countries where sex of the child contributed to more than 25% of the variability in U5MR, this factor was the only main contributor in Chad 2014/15, Côte d’Ivoire 2011/12, Ethiopia 2016, Gabon 2012, Malawi 2015/16, and Sierra Leone 2013 (Fig. 2).

#### Place of residence

The proportion of children living in urban areas varied from 9% in Burundi 2016/17 to 84% in Gabon 2012. After adjustment, the excess rural mortality was significant in Niger 2012, Nigeria 2013, Senegal 2010/11, and Comoros 2012, and the excess urban mortality was significant in Kenya 2014 and Tanzania 2015/16 (Table 2). Among the 8 countries where place of residence contributed to more than 25% of the variability in U5MR, this factor was the only main contributor in Kenya 2014, Niger 2012, Senegal 2010/11, Tanzania 2015/16, and Namibia 2013 (Fig. 2).

#### Mother’s education

The proportion of children whose mother had at least a primary education level varied from 6% in Niger 2012 to 86% in Zimbabwe 2015. After adjustment, the observed protective effect of mother’s education on U5MR was significant in Gambia 2013, Nigeria 2013, Togo 2013/14, Cameroon 2011, and Uganda 2016. Among the 12 countries where mother’s education contributed to more than 25% of the variability in U5MR, this factor was the only main contributor in Ghana 2014, Gambia 2013, Liberia 2013, and Togo 2013/14, four countries in Western Africa (Fig. 2).

#### Household wealth

After adjustment, the observed protective effect of household wealth on U5M was significant in 10 countries. Among the 11 countries where household wealth contributed to more than 25% of the variability in U5MR, this factor was the only main contributor in Angola 2015/16, Burkina Faso 2010, DR Congo 2013/14, Guinea 2012, Mali 2012/13, and Zimbabwe 2015 (Fig. 2).

#### Combination of socioeconomic factors

In some countries, more than one factor contributed to more than 25% of the variability in U5MR: sex of the child and mother’s education in Mozambique 2011, Uganda 2016, Zambia 2013/14, and Rwanda 2014/15; sex of the child and household wealth in Benin 2011/12, sex of the child and place of residence in Congo 2011/12; mother’s education and household wealth in Burundi 2016/17 and Nigeria 2013, household wealth and place of residence in Namibia 2013, mother’s education and place of residence in Comoros 2012 and Lesotho 2014, and sex of the child, mother’s education, and household wealth in Rwanda 2014/15.

## Discussion

### Main results

This study shows that major inequalities in U5MR still exist in sub-Saharan Africa in 2010–2016 but that there is not just one single story applicable to all countries. The relative contribution of four socioeconomic factors to the variability in U5MR was assessed using a regression-based decomposition of a Gini index.

The main contributors differed according to the country.

In Benin, Chad, Congo, Côte d’Ivoire, Ethiopia, Gabon, Malawi, Mozambique, Rwanda, Sierra Leone, Uganda, and Zambia, considerable inequalities between boys and girls were observed. It has been shown that differences in survival between boys and girls exist at least up to the age of 5 years [14]. An excess male child mortality can be explained by biological factors (lower resistance to infection, higher risk of premature birth, difficult labour related to a larger average body size and head circumference), gender discrimination (differential feeding and medical care practices, or response to HIV-related drugs) [22, 23]. After adjusting for a range of individual, household and community variables (including age, birth order, household wealth, maternal education but also skilled birth attendance and other factors), the excess male mortality remained significant in several countries in Sub-Saharan Africa [24].

In Comoros, Congo, Kenya, Lesotho, Namibia, Niger, Senegal, and Tanzania, considerable inequalities related to the place of residence were observed in this analysis. In Comoros, Lesotho, Namibia, Niger, and Senegal, U5MR was lower in urban areas while in Congo, Kenya, and Tanzania, U5MR was lower in rural areas. The effect of place of residence on U5MR, adjusted for sex of the child, mother’s education, and household wealth, may have operated through ecological setting, political economy and health system [13, 25]. Though the urban-rural difference is narrowing or even reversing in some countries (as a result of a more rapid mortality decline in rural areas than in urban areas and deplorable living conditions in urban slums [26]), an urban advantage persists in many countries. This urban advantage can be attributed to access to health services and better economic opportunities for families [27, 28]. In Niger, the observed excess infant mortality, was partly explained by the existence of a health facility within the community [29].

In Burundi, Cameroon, Comoros, Gambia, Ghana, Lesotho, Liberia, Mozambique, Nigeria, Rwanda, Uganda, and Zambia, considerable inequalities related to the mother’s educational level were observed, with lower mortality rates if the mother had at least a primary educational level. The effect of mother’s education on U5MR, adjusted for sex of the child, place of residence, and household wealth, may have operated through empowerment, health and reproductive behaviour (e.g. birth spacing) or health services utilization (e.g. knowledge, awareness) [13, 25]. This protective effect of maternal education adjusted for more covariates than in the study at hand, was also reported in Ghana [30], and Comoros [31]. Some assumptions suggested in a study on the factors associated with U5MR in rural Ghana were: educated mothers are more likely to receive antenatal care [32] (though the gap seems to be closing over time [33]), and motherhood could be delayed, decreasing the total number of children [34]. A recent study including, among other countries, Ghana and Nigeria showed that women’s education was associated with utilization of maternal health services: type of antenatal care provider, timing and frequency of antenatal care visits, place of delivery and presence of a skilled birth attendant at delivery [35]. Another study including Cameroon and Niger (among other countries), highlighted that the decline in under-five mortality rates, during last two decades, can be partly due to the government policies on women’s education, resulting in increased maternal awareness about child health and hygiene [36].

In Angola, Benin, Burundi, Burkina Faso, DR Congo, Guinea, Mali, Namibia, Nigeria, Rwanda, and Zimbabwe, considerable inequalities related to the household wealth were observed, with lower mortality rates in richest households. The effect of household wealth on U5MR, adjusted for sex of the child, place of residence, and mother’s educational level, may have operated through access to goods and services such as food, housing, transportation, or financial access to care [13, 25]. A study looking at changes in inequalities between the poorest and the least poor in mortality levels, using comparisons between successive surveys, showed that these inequalities widened in some of the afore-mentioned countries: Benin (2001), Namibia (2000) [37], Burundi (2010), Burkina Faso (2010), DR Congo (2007), Mali (2010), Nigeria (2008), and Rwanda (2010) [38].

The current paper focuses on U5MR but a sub-analysis by age group was also conducted (see Appendix 2), distinguishing between neonatal mortality (0–1 month), post-neonatal mortality (1–11 months), infant mortality (0–11 months) and child mortality (12–59 months). This analysis showed that the main sources of inequality vary according to the age group: household wealth and sex of the child for neonatal mortality, mother’s education and sex of the child for infant mortality, mother’s education, household wealth and place of residence for post-neonatal and child mortality. This is in line with previous literature showing changes in the sex ratios of mortality as children get older [14], and suggests that later in the life of children, the socioeconomic factors such as household wealth, mother’s education and place of residence, may become more important sources of inequalities between subgroups. We report here mainly on under− 5 mortality, because it is one of the indicators recommended by the World Health Organization for policy-oriented monitoring of equity (“Under-5 mortality and, where possible, its components assessed separately: neonatal, neonatal, postneonatal, and infant mortality, and mortality among children 1-4 years”) [39]. The small number of deaths in the subgroup 1–4 years made the analysis less reliable. Further analysis should also consider children aged between 5 and 14 years, because about 1 million die globally in this age group [40], but there is very limited research on socioeconomic factors of child mortality beyond age 5.

### Limitations

The DHS program provide quality survey data, both through internal quality assurance and control procedures (continuous instrument quality checks, appropriate field personnel training, high response rate [41] and through its transparent data files and survey methods descriptions [42]. However, the cross-sectional design of the survey does not allow accounting for the order of events; information on mother or household characteristics at the time of the interview does not fully reflect the status at child death. Information on child death depends on the mother’s will and ability to communicate the information, and reporting quality may vary across social groups [6]. Moreover, the U5MR estimates based on birth histories may differ from estimates based on vital registration systems, due to recall errors in reporting vital events or ages of children at death or at survey [43]. In addition, due to relatively small sample sizes, some categories have to been pooled together. In this study, mother’s education “secondary or higher” was pooled with primary education because too few cases were present in the former category, resulting in aberrant estimates. This type of transformation is likely to reduce the explanatory power of this variable. Similarly, household wealth tertile was dichotomized to avoid overestimating its contribution. In Sierra Leone, the middle wealth tertile showed the highest U5MR while in Comoros, the middle wealth tertile showed the lowest U5MR, but these differences were not significant. These effects were not shown in the analyses presented above and focusing on a difference between the richest and the others. Finally, it should be kept in mind that the four factors are hierarchically related [44] and the effect of place of residence may have been mediated by mother’s education or household wealth, thereby underestimating its relative contribution.

The study focuses on four known socioeconomic factors, but some external factors, such as conflicts, with potentially large impact on child mortality and socioeconomic resources in a country, were not adjusted for. A study matching birth histories from Demographic and Health Surveys with data on proximity to armed conflict (reported in the Uppsala Conflict Data Program Georeferenced Events Dataset) showed that the risk of dying before reaching age 1 was 5·2 per 1000 births higher for a child born within 50 km of an armed conflict than for a child born in the same region during periods without conflict (corresponding to a 7·7% increase above baseline) [45]. Among the 32 countries included in this study, 11 countries were affected by an armed conflict during the survey or in the five preceding years. The most affected countries were DR Congo (6617 armed-related deaths during the defined period), Nigeria (3169 deaths), and Mali (1349 deaths) [46]. However, studying how the socioeconomic factors contributing to under-five mortality in Sub-Saharan Africa change in conflict-affected settings is beyond the scope of this paper. More broadly, any positive or negative change in the organization or resources of a country or a community (health insurance, epidemics, pollution, migration or fertility) is likely to impact the distribution of the socioeconomic resources and their combined effect on child health.

### Perspectives

First, the study focuses on relative inequality in U5MR between subgroups of the population. Considering absolute differences between groups is as important [47] because a low relative difference may hide a high absolute difference in high-mortality countries. The use of multivariate additive hazard models, also combining the prevalence and impact of the factors, [48, 49] could allow balancing the importance in absolute differences of each factor controlling for others, with a possibility to consider the “background” contribution of the factors not included.

Second, the identification of the main socioeconomic contributors to the variability in U5MR among the four factors deserve more investigations. To this end, other known factors associated with U5MR (such as antenatal care, skilled attendance at birth, access to sanitation facilities, age of the mother at birth, parity, region etc.) could be added in the multivariate models in order to explain the observed differences (but in DHS, some of these factors are known only for the surviving children). In addition, if a wealth-related inequality in U5MR is concerning, a regression-based decomposition of the concentration index could be performed, as was done before [11].

Third, another way forward would be to assess the association between changes in the socioeconomic factors and changes in U5MR over time, as Demographic and Health surveys, are being conducted every 5 years and allow exploring the impact of possible changes in the socioeconomic resources induced by major policies or crises in a country.

Fourth, although we found no significant correlation between U5MR and the Gini index related to sex of the child, place of residence, mother’s education or household wealth, this observation does not exclude a correlation with the Gini index commonly used to measure income-related inequality in a country [46]. Possible correlations between indicators such as U5MR, the “traditional Gini Index”, “Gross Domestic Product”, and other country-level indicators could be done using the data provided with the World Bank. Such an ecological (country-level) study was conducted on under-five mortality trends in sub-Saharan Africa between 1960 and 2000 and showed that U5MR was negatively associated with per capita income and urbanization, and positively associated with illiteracy [50]. Another study showed that the decline in U5MR was associated with several Worldwide Governance Indicators: government effectiveness, rule of law, control of corruption, regulatory quality, political stability and absence of violence [38].

Finally, in this study, the independent effect of the four socioeconomic factors on U5MR determined their contributions to the variability in U5MR. However, these factors may have interacting effects and the major differences could be between subgroups defined by several dimensions (e.g. girls from richest households living in urban areas vs boys from poorest households living in rural areas). A more in-depth detection of inequalities could be studied by including interaction terms into the regression models, or by using tools handling unspecified complex interactions such as classification and regression trees [51] and random forest [52].

## Conclusions

Socioeconomic inequalities in U5MR exist in all countries, but the socioeconomic dimensions may differ across countries. Identifying these main contributors is important to guide research and interventions aiming at reducing U5MR in all population subgroups. Mother’s educational level appeared as an important factor in a majority of countries, followed by sex of the child, household wealth, and place of residence.

## Figures and Tables

**Fig. 3:**
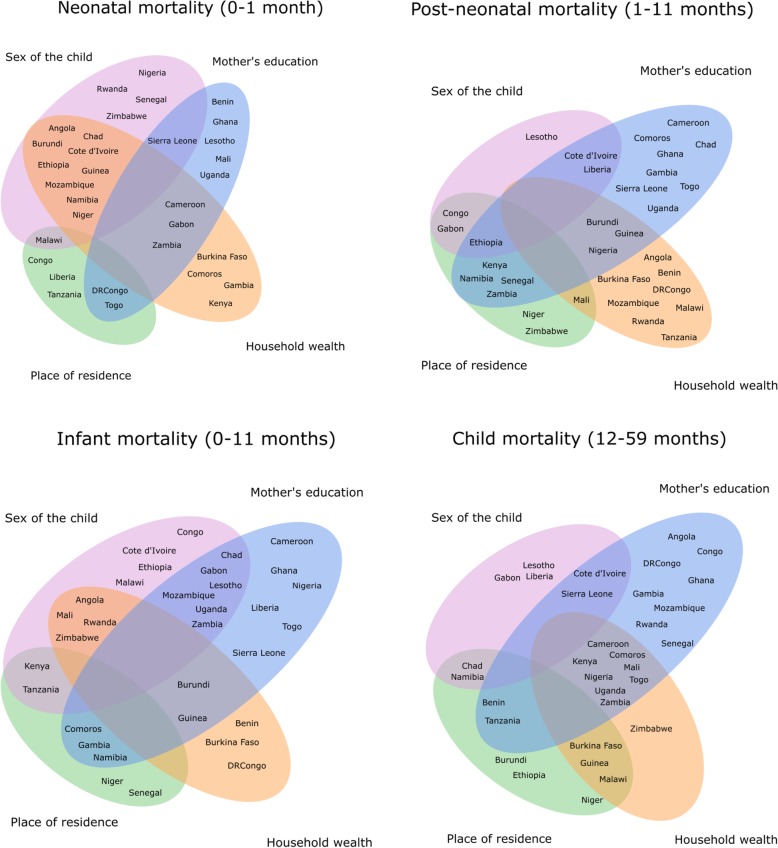
Classification of the 32 countries according to the main contributors to the variability in U5M. A factor was defined as a main contributor if its contribution to the variability in U5M exceeded 25% according to the regression-based decomposition of the Gini index (Tables)

## Data Availability

The data that support the findings of this study are available from the Measure DHS program (https://dhsprogram.com/) but restrictions apply to the availability of these data, which were used under license for the current study, and so are not publicly available. Data are however available from the authors upon reasonable request and with permission of the Measure DHS program.

## Appendix 1

### Overview of the R code used

# packages requiredlibrary(survey);library(foreign);library(car); library(party) ; library(decomp) ; library(VennDiagram)# import data and specify the survey designBenin<-read.dta("Benin.dta")Beninc<-svydesign(id=as.formula("~PSU+household"), weight=as.formula("~weights"), strata=as.formula("~region"),data=Benin)# Poisson regression-based decomposition methodm<-svyglm(deadu5~female+rural+ed+richest,offset=log(time),Beninc, family="quasipoisson")pr <- as.numeric(predict(m, newdata = m$model[, -1],vcov = FALSE))Benin$pr<-prc<-contribution(m,Benin$pr)# Venneuler (csv file one column by determinant coded 1/0 important or not)d<-read.csv("Imp.csv", sep=";", header=T)venn.diagram(x = list(Sex = which(d[,1]==1),Residence = which(d[,2]==1),Education = which(d[,3]==1),Wealth = which(d[,4]==1)),imagetype = "svg",fill = c("plum", "palegreen3", "cornflowerblue", "tan1"), filename="venn.svg");# Figure 1 map, example for U5Mlibrary(maptools) ; library(sp) ; library(lattice) ; library(shapefiles)library(RColorBrewer) ; library(classInt)shp<-readShapePoly("AfricanCountires")shp=merge(shp,result,by='COUNTRY', all.x=T, duplicateGeoms=T)quantile(shp$U5M[shp$U5M>0], probs = (0:5)/5)brks<-classIntervals(shp$U5M,n=5,style="fixed", fixedBreaks=c(0,49,63,68.8,93,119.2,152))cols<-brewer.pal(6, "Reds")plot(shp, col=cols[findInterval(shp$U5M, brks$brks, all.inside=T)])legend("bottomleft", legend=leglabs(round(brks$brks,1), under="<", over=">", between="-"),fill=cols, title= "Under-five mortality (‰)", inset = .12, cex=0.8)

## Appendix 2

### Analysis by age group

**Table 3:** Neonatal mortality (0–1 month)

				Sex of the child				Place of residence				Mother’s education				Household wealth			
				Female				Urban				Primary+				Richest			
	n	G	mean(y)	mean	b	c	%_Gini_	mean	b	c	%_Gini_	mean	b	c	%_Gini_	mean	b	c	%_Gini_
Angola 2015–16	445	−.213	−.32	.41	−.15	−.45	40	.51	.01	−.07	0	.31	.12	.07	4	.29	**−.26**	−.51	56
Benin 2011–12	406	−.065	−.28	.43	.01	.22	6	.43	.00	−.05	0	.13	.12	.87	73	.34	−.03	−.35	21
Burkina Faso 2010	560	−.102	−.29	.44	.03	.32	13	.15	−.07	−.76	25	.06	.08	−.17	0	.29	−.09	−.69	62
Burundi 2016–17	395	−.121	−.31	.46	−.07	−.33	29	.12	−.05	.04	0	.26	−.09	−.36	23	.34	.12	.44	48
Cameroon 2011	434	−.090	−.22	.49	−.03	−.21	14	.38	−.02	.38	0	.44	.03	.48	32	.29	.06	.69	55
Chad 2014–15	712	−.192	−.24	.44	**−.10**	−.42	42	.20	**−.13**	−.02	1	.16	−.09	−.39	12	.31	**.17**	.38	45
Comoros 2012	100	−.255	−.35	.43	−.03	−.17	2	.19	−.10	−.23	5	.33	.06	.33	7	.36	.33	.64	85
Congo 2011–12	250	−.173	−.29	.38	.01	.01	0	.64	**.20**	.36	90	.71	.03	.24	10	.30	−.01	.02	0
Cote d’Ivoire 2011–12	367	−.122	−.21	.30	−.07	−.66	51	.37	−.07	.16	0	.16	.06	.42	15	.37	.07	.39	34
DRCongo 2013–14	711	−.114	−.30	.47	−.05	−.28	20	.29	−.12	−.55	53	.48	.10	.20	27	.31	.01	−.26	0
Ethiopia 2016	419	−.232	−.30	.33	**−.24**	−.57	63	.12	.09	.26	4	.10	−.10	−.20	3	.38	.16	.34	30
Gabon 2012	209	−.152	−.38	.42	.03	.18	4	.87	−.10	−.07	11	.66	−.13	−.19	27	.24	.21	.66	58
Gambia 2013	251	−.214	−.36	.45	.04	.10	2	.51	−.09	.15	0	.29	−.16	−.19	10	.33	**.34**	.67	87
Ghana 2014	208	−.139	−.22	.43	.04	.36	22	.45	−.04	−.09	5	.55	.09	.45	69	.35	.03	.12	4
Guinea 2012	298	−.119	−.24	.46	−.06	−.39	37	.23	.08	−.09	0	.08	−.05	−.59	9	.31	−.11	−.47	54
Kenya 2014	609	−.108	−.30	.46	.02	.15	4	.41	.07	−.06	0	.60	.07	.05	6	.36	−.16	−.54	90
Lesotho 2014	135	−.234	−.21	.45	.00	.00	0	.18	−.02	−.11	1	.70	**−.20**	−.30	83	.26	.14	.22	16
Liberia 2013	276	−.138	−.23	.46	.03	.18	8	.49	.12	.51	84	.29	.03	.34	8	.34	−.05	.26	0
Malawi 2015–16	586	−.174	−.25	.41	**−.12**	−.50	54	.14	**.19**	.70	41	.31	−.02	−.09	1	.34	−.07	−.05	3
Mali 2012–13	437	−.178	−.22	.39	−.06	−.30	18	.14	.16	.45	25	.08	−.24	−.92	47	.26	−.08	−.18	10
Mozambique 2011	441	−.168	−.26	.43	−.11	−.40	41	.29	.03	.41	8	.15	−.08	−.01	0	.35	.12	.55	50
Namibia 2013	136	−.126	−.30	.41	−.07	−.39	30	.45	.02	.27	6	.80	−.09	−.11	21	.29	.09	.61	44
Niger 2012	422	−.142	−.39	.44	−.15	−.40	49	.10	−.16	−.08	2	.04	−.09	−.35	2	.29	**.17**	.51	46
Nigeria 2013	1465	−.137	−.21	.45	**−.08**	−.55	72	.31	−.06	−.33	21	.40	.00	−.03	0	.29	−.02	−.31	7
Rwanda 2014–15	227	−.180	−.38	.48	**−.22**	−.52	81	.14	.14	.47	14	.34	−.02	−.11	1	.34	−.11	−.07	4
Senegal 2010–11	476	−.105	−.26	.38	−.10	−.60	79	.31	.07	.28	21	.11	.00	.26	0	.29	−.03	.03	0
Sierra Leone 2013	562	−.119	−.24	.47	−.08	−.47	63	.30	−.03	−.02	1	.26	−.07	−.42	27	.37	.05	.14	10
Tanzania 2015–16	355	−.238	−.32	.44	−.12	−.30	17	.36	**.33**	.64	82	.74	−.04	−.02	1	.42	−.14	.27	0
Togo 2013–14	224	−.141	−.21	.41	−.06	−.19	12	.33	.18	.37	55	.26	−.10	−.51	33	.33	−.12	.23	0
Uganda 2016	574	−.141	−.34	.45	−.08	−.30	22	.20	.09	.28	11	.44	**−.15**	−.47	66	.37	.03	.03	1
Zambia 2013–14	436	−.089	−.29	.42	−.02	−.33	9	.32	.03	.40	15	.49	−.08	−.25	41	.34	.07	.38	35
Zimbabwe 2015	206	−.234	−.22	.46	−.17	−.54	81	.29	−.07	−.29	12	.84	−.09	−.05	7	.28	.02	−.27	0

G: Gini index; mean(y): mean of the predicted (ln) death rates; mean: mean of the dummy variable (coded 0/1), corresponds to the proportion of the factor in the country; b: regression coefficient from a multivariate Poisson model (in bold if significant); %_Gini_: relative factor’s contribution calculated with the regression-based decomposition of the Gini index_h_; A few negative contributions were restricted to 0

**Table 4:** Post-neonatal mortality (1–11 months)

				Sex of the child				Place of residence				Mother’s education				Household wealth			
				Female				Urban				Primary+				Richest			
	n	G	mean(y)	mean	b	c	%_Gini_	mean	b	c	%_Gini_	mean	b	c	%_Gini_	mean	b	c	%_Gini_
Angola 2015–16	3069	−.042	−4.48	.50	−.10	−.26	7	.59	−.10	−.34	11	.39	−.04	−.45	4	.33	**−.68**	−.67	79
Benin 2011–12	2762	−.050	− 4.5	.48	**−.27**	−.26	14	.41	.11	−.21	0	.18	−.07	−.49	3	.34	**−.87**	−.66	83
Burkina Faso 2010	3264	−.036	−3.87	.50	−.05	−.24	4	.16	−.07	−.78	6	.07	−.36	−.84	16	.33	**−.47**	−.67	74
Burundi 2016–17	2684	−.047	−4.26	.48	−.01	−.20	0	.10	.43	−.43	0	.26	**−.45**	−.63	34	.33	**−.67**	−.66	65
Cameroon 2011	2603	−.042	−4.07	.49	−.14	−.16	7	.43	−.10	−.39	10	.47	**−.49**	−.53	71	.33	−.12	−.55	12
Chad 2014–15	3957	−.033	−3.77	.48	−.14	−.38	20	.21	.12	.07	1	.17	**−.67**	−.83	78	.33	−.01	−.11	0
Comoros 2012	658	−.093	−5	.51	.20	.15	3	.27	−.56	−.58	19	.35	**−1.60**	−.65	78	.36	.06	−.13	0
Congo 2011–12	2056	−.022	−4.59	.51	−.29	−.47	68	.61	−.21	−.22	28	.72	.09	.01	1	.34	−.04	−.29	4
Cote d’Ivoire 2011–12	1763	−.035	−4.06	.50	**−.37**	−.39	50	.36	−.07	−.36	7	.19	−.39	−.76	38	.33	−.07	−.37	6
DRCongo 2013–14	4102	−.025	−4.04	.50	.05	.14	3	.30	.26	−.25	0	.50	−.17	−.36	24	.33	**−.43**	−.63	73
Ethiopia 2016	2120	−.043	−4.47	.51	−.32	−.32	28	.12	−.68	−.83	35	.12	−.72	−.86	37	.35	.02	−.08	0
Gabon 2012	1352	−.029	−4.82	.48	−.33	−.37	42	.84	**−.58**	−.16	56	.77	−.03	−.12	2	.35	.03	.00	0
Gambia 2013	1826	−.032	−4.89	.52	−.07	−.14	3	.46	.37	.11	12	.33	−.59	−.67	85	.31	.03	.05	0
Ghana 2014	1239	−.028	−4.87	.47	−.01	−.13	1	.45	.28	.11	10	.59	−.51	−.41	89	.32	.02	.03	0
Guinea 2012	1579	−.063	−4.01	.48	−.11	−.22	4	.26	.28	−.51	0	.14	**−.78**	−.82	30	.32	**−.94**	−.64	66
Kenya 2014	4028	−.024	−4.5	.50	−.25	−.22	25	.35	.30	.30	29	.61	−.29	−.28	46	.32	−.02	−.04	0
Lesotho 2014	740	−.051	−4.29	.53	−.42	−.31	32	.26	.40	.49	23	.82	−.51	−.11	20	.31	.40	.43	24
Liberia 2013	1712	−.025	−4.21	.47	.21	.35	32	.49	.18	.01	1	.32	−.30	−.62	56	.31	−.17	−.25	12
Malawi 2015–16	3296	−.018	−4.62	.48	.00	.16	0	.14	−.15	−.82	20	.30	.02	−.05	0	.33	−.30	−.67	80
Mali 2012–13	2171	−.039	−4.33	.49	−.01	−.21	1	.20	−.43	−.79	40	.10	−.04	−.74	2	.35	−.44	−.65	58
Mozambique 2011	2474	−.031	−4.03	.49	.10	.20	8	.27	−.14	−.63	20	.18	−.19	−.74	21	.32	−.29	−.67	52
Namibia 2013	1111	−.036	−4.52	.51	.26	.30	23	.45	−.42	−.43	47	.78	−.40	−.16	30	.29	.25	−.14	0
Niger 2012	2694	−.024	−4.14	.48	.10	.37	18	.13	**−.65**	−.87	73	.06	.20	.10	1	.33	−.05	−.40	7
Nigeria 2013	6797	−.046	−4.07	.51	−.14	−.18	7	.35	−.06	−.47	5	.46	**−.44**	−.53	58	.33	−.27	−.62	30
Rwanda 2014–15	1632	−.047	−4.93	.50	−.28	−.28	16	.18	.23	−.37	0	.36	−.24	−.38	13	.35	**−.77**	−.66	71
Senegal 2010–11	2600	−.046	−4.66	.48	.27	.23	14	.37	**−.45**	−.58	44	.11	−.80	−.87	36	.32	−.08	−.48	6
Sierra Leone 2013	2753	−.019	−3.5	.49	.06	.21	10	.25	.00	−.54	0	.24	**−.26**	−.76	70	.33	−.08	−.55	20
Tanzania 2015–16	2038	−.006	−4.45	.49	−.02	−.17	4	.27	.02	−.29	0	.67	.07	.15	25	.32	−.11	−.60	71
Togo 2013–14	1433	−.032	−4.35	.48	.01	.17	0	.34	−.07	−.52	9	.31	**−.52**	−.69	81	.31	−.08	−.56	10
Uganda 2016	3089	−.055	−4.65	.50	**−.33**	−.26	16	.19	−.10	−.28	2	.42	**−.89**	−.58	82	.32	.22	−.16	0
Zambia 2013–14	2621	−.020	−4.34	.48	−.13	−.21	16	.35	.35	.30	42	.52	−.19	−.34	40	.34	−.19	−.03	2
Zimbabwe 2015	1205	−.024	−4.3	.50	−.08	−.22	9	.26	−.24	−.73	44	.85	−.18	−.15	22	.29	−.13	−.71	25

G: Gini index; mean(y): mean of the predicted (ln) death rates; mean: mean of the dummy variable (coded 0/1), corresponds to the proportion of the factor in the country; b: regression coefficient from a multivariate Poisson model (in bold if significant); %_Gini_: relative factor’s contribution calculated with the regression-based decomposition of the Gini index_h_; A few negative contributions were restricted to 0

**Table 5:** Infant mortality (0–11 months)

				Sex of the child				Place of residence				Mother’s education				Household wealth			
				Female				Urban				Primary+				Richest			
	n	G	mean(y)	mean	b	c	%_Gini_	mean	b	c	%_Gini_	mean	b	c	%_Gini_	mean	b	c	%_Gini_
Angola 2015–16	3514	−.047	−3.61	.49	**−.31**	−.31	28	.58	−.20	−.32	22	.38	−.06	−.42	6	.32	**−.35**	−.66	44
Benin 2011–12	3168	−.033	− 3.61	.47	**−.21**	−.24	19	.41	.12	−.20	0	.17	−.23	−.75	23	.34	**−.35**	−.63	58
Burkina Faso 2010	3824	−.035	−3.26	.49	−.12	−.27	13	.16	−.01	−.69	1	.07	−.24	−.84	13	.32	**−.38**	−.68	73
Burundi 2016–17	3079	−.027	− 3.51	.48	−.08	−.20	9	.10	.32	.18	6	.26	**−.30**	−.69	59	.33	−.18	−.42	26
Cameroon 2011	3037	−.030	−3.31	.49	−.09	−.13	6	.42	−.13	−.47	25	.46	**−.23**	−.53	55	.32	−.08	−.56	14
Chad 2014–15	4669	−.031	−3.11	.47	**−.20**	−.39	37	.21	.05	.08	1	.17	**−.41**	−.83	61	.33	.02	.13	1
Comoros 2012	758	−.051	−3.72	.50	−.12	−.19	6	.26	−.59	−.72	57	.35	−.36	−.49	33	.36	.32	.06	4
Congo 2011–12	2306	−.037	−3.75	.50	**−.45**	−.50	81	.61	.18	.08	7	.72	.04	.08	2	.33	−.21	−.21	10
Cote d’Ivoire 2011–12	2130	−.059	−3.22	.47	**−.64**	−.54	85	.36	−.09	−.01	0	.18	−.26	−.45	11	.34	.16	.13	4
DRCongo 2013–14	4813	−.018	−3.34	.50	−.07	−.25	13	.30	.08	−.33	0	.49	−.02	−.25	3	.33	−.26	−.67	84
Ethiopia 2016	2539	−.062	−3.48	.48	**−.71**	−.52	83	.12	−.18	−.12	1	.11	−.46	−.43	11	.35	.21	.16	6
Gabon 2012	1561	−.041	−3.75	.47	−.26	−.36	30	.84	.03	−.02	0	.75	**−.45**	−.24	52	.33	−.23	−.36	18
Gambia 2013	2077	−.036	−3.83	.51	−.15	−.20	11	.46	.30	.36	37	.32	−.43	−.33	33	.31	.18	.44	18
Ghana 2014	1447	−.022	−3.62	.47	−.08	−.29	13	.45	−.06	.04	0	.59	−.28	−.32	64	.32	.27	.22	23
Guinea 2012	1877	−.047	−3.23	.47	−.10	−.24	7	.26	.02	−.53	0	.13	**−.60**	−.87	43	.32	−.38	−.62	49
Kenya 2014	4637	−.025	−3.62	.49	**−.21**	−.32	36	.36	**.29**	.50	55	.61	−.17	−.08	9	.33	−.05	.17	0
Lesotho 2014	875	−.055	−3.38	.52	−.39	−.34	37	.25	−.04	−.05	0	.80	**−.71**	−.18	55	.30	.33	.15	8
Liberia 2013	1988	−.019	−3.47	.47	.12	.30	25	.49	.11	.09	7	.32	−.23	−.60	65	.31	.07	.09	3
Malawi 2015–16	3882	−.018	−3.54	.47	**−.22**	−.53	83	.14	.12	.43	11	.30	−.01	−.11	0	.33	−.11	−.12	6
Mali 2012–13	2608	−.047	−3.33	.48	**−.29**	−.29	26	.19	−.08	−.70	7	.10	−.16	−.62	6	.33	**−.44**	−.66	61
Mozambique 2011	2915	−.020	−3.3	.48	−.14	−.37	37	.28	.00	−.22	0	.17	**−.29**	−.83	63	.33	.02	−.07	0
Namibia 2013	1247	−.020	−3.73	.50	−.11	−.22	16	.45	−.20	−.38	44	.78	−.21	−.18	40	.29	.16	−.03	0
Niger 2012	3116	−.022	−3.46	.48	−.08	−.42	22	.12	**−.52**	−.88	74	.06	−.07	−.62	3	.32	.02	−.07	0
Nigeria 2013	8262	−.044	− 3.2	.50	**−.24**	−.22	19	.34	−.14	−.50	17	.45	**−.30**	−.53	51	.32	−.10	−.58	14
Rwanda 2014–15	1859	−.032	−3.9	.50	**−.32**	−.40	52	.17	.02	−.29	0	.36	−.15	−.40	17	.34	−.23	−.49	31
Senegal 2010–11	3076	−.031	−3.54	.47	−.19	−.31	25	.36	−.28	−.64	58	.11	−.10	−.51	5	.31	−.08	−.50	12
Sierra Leone 2013	3315	−.015	−2.93	.49	−.03	−.15	4	.26	.07	.19	7	.25	−.20	−.75	84	.34	.03	.20	4
Tanzania 2015–16	2393	−.034	−3.53	.48	−.20	−.33	27	.28	**.36**	.72	60	.68	.11	.14	9	.34	.03	.50	4
Togo 2013–14	1657	−.033	−3.47	.47	−.16	−.30	19	.34	−.14	−.07	3	.31	**−.43**	−.69	78	.32	.20	−.03	0
Uganda 2016	3663	−.039	−3.58	.49	**−.31**	−.37	41	.19	−.04	−.15	1	.42	**−.42**	−.45	57	.33	**.26**	.02	1
Zambia 2013–14	3057	−.021	−3.51	.47	**−.22**	−.53	73	.34	.00	.02	0	.51	−.15	−.26	27	.34	.06	.00	0
Zimbabwe 2015	1411	−.024	−3.36	.49	−.19	−.43	36	.27	.35	−.34	0	.85	−.14	−.09	10	.29	−.43	−.49	54

G: Gini index; mean(y): mean of the predicted (ln) death rates; mean: mean of the dummy variable (coded 0/1), corresponds to the proportion of the factor in the country; b: regression coefficient from a multivariate Poisson model (in bold if significant); %_Gini_: relative factor’s contribution calculated with the regression-based decomposition of the Gini index_h_; A few negative contributions were restricted to 0

**Table 6:** Child mortality (11–59 months)

				Sex of the child				Place of residence				Mother’s education				Household wealth			
				Female				Urban				Primary+				Richest			
	n	G	mean(y)	mean	b	c	%_Gini_	mean	b	c	%_Gini_	mean	b	c	%_Gini_	mean	b	c	%_Gini_
Angola 2015–16	10,805	−.025	−7.94	.51	−.01	−.11	0	.61	.14	−.12	0	.37	−.70	−.63	79	.34	−.22	−.59	21
Benin 2011–12	10,239	−.035	− 7.79	.49	−.10	−.17	3	.39	**−.60**	−.58	50	.14	−.63	−.79	26	.33	−.30	−.61	22
Burkina Faso 2010	11,215	−.026	−6.86	.49	−.11	−.22	7	.17	**−.45**	−.81	35	.07	−.64	−.90	24	.34	**−.30**	−.63	35
Burundi 2016–17	10,110	−.022	−7.63	.50	−.10	−.19	6	.09	**−1.35**	−.91	67	.25	−.16	−.66	16	.33	−.10	−.58	12
Cameroon 2011	8695	−.052	−6.95	.51	.21	.17	5	.42	−.18	−.41	9	.48	**−.85**	−.51	58	.34	**−.51**	−.60	28
Chad 2014–15	13,947	−.012	−6.78	.49	−.18	−.36	42	.19	.26	.49	32	.14	.11	.37	7	.33	**−.26**	−.17	19
Comoros 2012	2380	−.037	−8.5	.49	.01	.13	0	.27	−.39	−.48	16	.35	−.79	−.62	54	.33	−.50	−.56	29
Congo 2011–12	7022	−.021	−7.55	.51	.24	.28	22	.60	.38	.07	10	.70	−.48	−.27	58	.33	−.21	−.22	10
Cote d’Ivoire 2011–12	5645	−.028	−7.22	.51	−.41	−.35	36	.38	−.17	−.32	10	.18	**−.74**	−.82	54	.33	.03	−.24	0
DRCongo 2013–14	13,901	−.018	−7.18	.50	−.05	−.13	3	.31	.07	−.38	0	.47	**−.41**	−.53	76	.33	−.16	−.54	21
Ethiopia 2016	8099	−.030	−8.05	.48	.01	.25	1	.11	**−2.03**	−.89	81	.08	−.42	−.87	13	.33	.22	.20	6
Gabon 2012	4502	−.037	−7.65	.48	−.77	−.50	66	.84	−.52	−.04	6	.74	−.21	−.06	4	.33	.59	.35	24
Gambia 2013	6009	−.034	−8.29	.49	.23	.17	7	.48	.23	.13	5	.32	**−1.15**	−.68	87	.34	.14	.06	1
Ghana 2014	4435	−.065	−8.75	.48	−.35	−.16	5	.45	−.25	−.34	7	.58	**−1.77**	−.42	76	.34	−.38	−.56	13
Guinea 2012	5161	−.035	−7.02	.49	−.11	−.24	6	.26	−.39	−.73	31	.12	−.10	−.60	3	.34	**−.67**	−.66	61
Kenya 2014	16,323	−.021	−8.33	.49	.15	.12	5	.36	.46	−.11	0	.59	−.28	−.30	26	.34	−.64	−.62	69
Lesotho 2014	2263	−.033	−7.72	.50	**.96**	.50	94	.30	.10	.16	2	.78	−.11	−.12	4	.35	.01	.12	0
Liberia 2013	5618	−.003	−7.2	.49	−.06	−.50	68	.50	.03	.25	18	.32	.00	.05	0	.34	.03	.32	14
Malawi 2015–16	13,401	−.017	−8.09	.51	.14	.25	13	.13	−.46	−.86	38	.29	−.10	−.43	9	.33	−.26	−.65	41
Mali 2012–13	7717	−.043	−7.47	.49	**−.38**	−.26	15	.19	−.12	−.68	5	.09	**−1.50**	−.91	40	.33	−.60	−.64	40
Mozambique 2011	8187	−.007	−7.44	.50	−.07	−.29	20	.28	−.04	−.52	11	.16	−.19	−.84	51	.34	−.05	−.51	18
Namibia 2013	3798	−.022	−8.1	.52	.35	.28	29	.50	.58	.29	47	.77	−.21	−.09	9	.35	−.38	−.20	15
Niger 2012	9420	−.021	−6.7	.50	.04	.23	4	.13	**−.99**	−.87	82	.05	−.35	−.85	12	.34	.10	.12	3
Nigeria 2013	23,219	−.048	−7.05	.49	−.05	−.13	1	.35	**−.39**	−.55	23	.45	**−.48**	−.51	33	.34	**−.66**	−.66	44
Rwanda 2014–15	5995	−.037	−8.34	.49	−.07	−.17	2	.17	.02	−.35	0	.33	**−1.26**	−.67	90	.33	−.14	−.53	8
Senegal 2010–11	9250	−.187	−9.81	.49	−.06	−.22	0	.39	**−1.03**	−.56	12	.12	**−15.5**	−.88	87	.34	.02	−.31	0
Sierra Leone 2013	8622	−.017	−6.83	.51	**−.24**	−.33	34	.25	−.01	−.12	0	.20	**−.47**	−.80	64	.33	.15	.04	2
Tanzania 2015–16	7838	−.024	−7.8	.50	−.01	−.13	0	.27	**.83**	.33	40	.65	**−.48**	−.30	51	.33	−.49	−.11	10
Togo 2013–14	5320	−.035	−7.46	.50	−.01	−.14	0	.36	.03	−.47	0	.30	−.54	−.56	34	.34	−.78	−.66	65
Uganda 2016	11,858	−.036	−8.03	.50	−.24	−.15	6	.22	−.32	−.64	15	.41	−.39	−.49	27	.34	**−.67**	−.66	51
Zambia 2013–14	10,389	−.019	−7.64	.50	−.04	−.13	2	.34	.23	−.33	0	.49	−.38	−.49	54	.33	−.39	−.59	44
Zimbabwe 2015	4721	−.024	−7.99	.51	.00	.16	0	.33	.72	−.52	0	.86	−.42	−.12	14	.35	**−1.22**	−.65	86

Senegal: this analysis is not reliable (aberrant value for the regression coefficient of mother’s education) because no death occurred in the sub-group of women with an educational level of at least primary

G: Gini index; mean(y): mean of the predicted (ln) death rates; mean: mean of the dummy variable (coded 0/1), corresponds to the proportion of the factor in the country; b: regression coefficient from a multivariate Poisson model (in bold if significant); %_Gini_: relative factor’s contribution calculated with the regression-based decomposition of the Gini index_h_; A few negative contributions were restricted to 0

## Abbreviations

DHS: Demographic and Health Survey
DR Congo: the Democratic Republic of the Congo
SDG3: The third Sustainable Development Goal
U5MR: Under-five mortality rate
